# Supplementary material for: Ultra-Highly Efficient Removal of Methylene Blue Based on Graphene Oxide/TiO2/Bentonite Sponge
Source: Materials (Basel). 2020 Feb 11;13(4):824. doi: 10.3390/ma13040824 (PMC7078707; doi:10.3390/ma13040824)
Supplement: Supplementary file 1 [file materials-13-00824-s001.pdf]

materials

Supplementary Materials

Ultra-Highly Efficient Removal of Methylene Blue Based on Graphene Oxide/TiO_2/Bentonite Sponge

Yuan Liu [1], Luyan Wang [1,*], Ni Xue [2], Pengxiang Wang [1], Meishan Pei [1] and Wenjuan Guo [3]

1 School of chemistry and chemical Engineering, University of Jinan, Jinan 250022, China; 20172120450@mail.ujn.edu.cn (Y.L.); 20172120409@mail.ujn.edu.cn (P.W.); chm_peims@ujn.edu.cn (M.P.)
2 State Key Laboratory of Crystal Materials, Shandong University, Jinan 250100, China; 201820319@mail.sdu.edu.cn
3 Institute of Surface Analysis and Chemical Biology, University of Jinan, Jinan 250022, China; chm_guowj@ujn.edu.cn
* Correspondence: chm_wangly@ujn.edu.cn; Tel.: +86-531-89736800

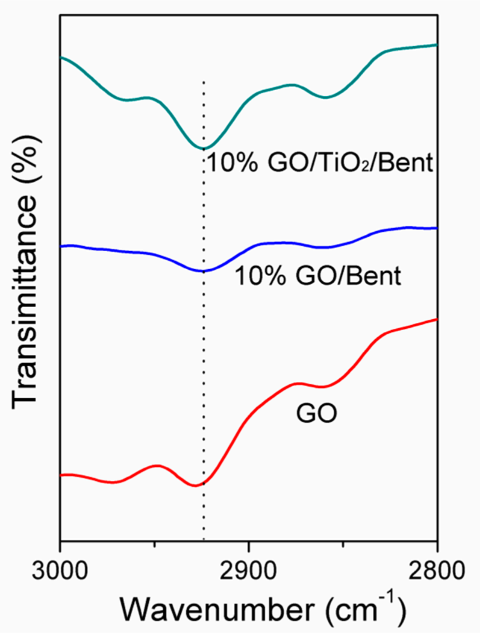

Figure S1. FTIR spectra of GO, 10% GO/Bent and 10% GO/TiO_2/Bent sponges.

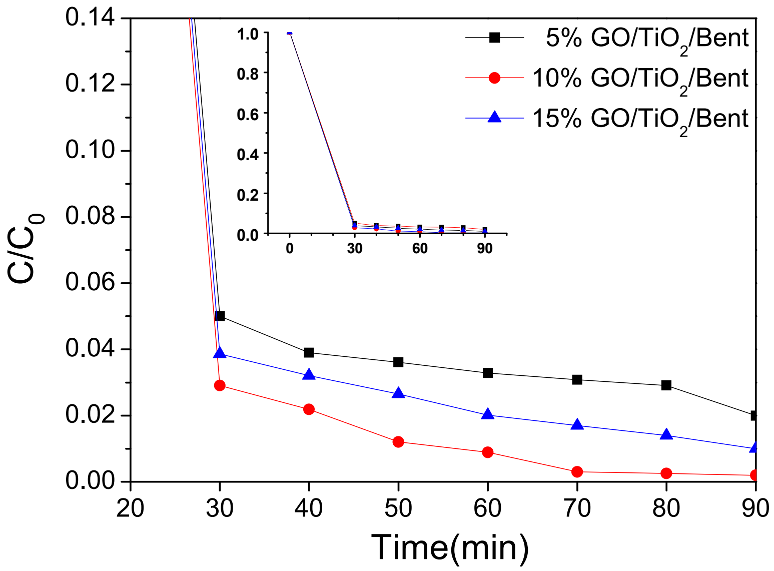

Figure S2. Photodegradation of MB by $GO/TiO_2/Bent$ sponge with different GO proportions under simulated sunlight irradiation after 30 min dark adsorption.

The effect of the graphene oxide (GO) content has been investigated on the photocatalytic efficiency of sponges under simulated sunlight irradiation for degradation of MB. It can be seen from Figure S1 that 10% $GO/TiO_2/Bent$ exhibits the highest photocatalytic efficiency. 5% $GO/TiO_2/Bent$ presents the lowest efficiency compared with others. This is probably because the amount of GO and Bent is too low to promote effectively the photocatalytic capacity of TiO_2. 15% $GO/TiO_2/Bent$ doesn't present the best probably due to the blockage of light by GO and Bent to TiO_2 surface [1]. Zhang et al. investigated MB degradation on GO/TiO_2 and also found that the higher addition of graphene in GO/TiO_2 leads to a decreased photocatalytic activity [2].

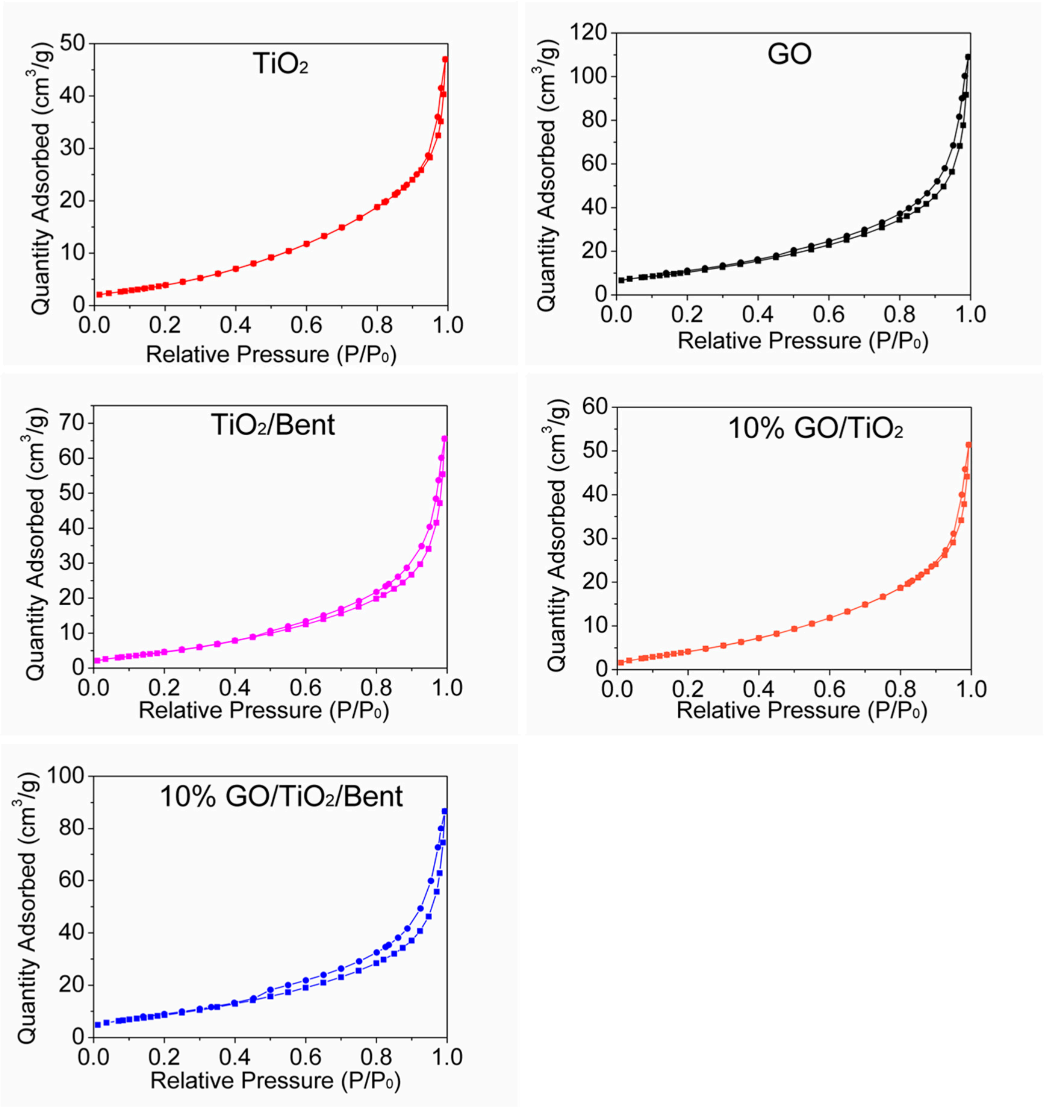

Figure S3. The N_2 adsorption-desorption isotherms of TiO_2, GO, TiO_2/Bent, 10% GO/TiO_2 and 10% GO/TiO_2/Bent.

Table S1. The BET surface area of GO, TiO_2, TiO_2/Bent, 10% GO/TiO_2 and 10% GO/TiO_2/Bent.

Sample	BET Surface Area (m^2/g)
TiO_2	17.80
GO	40.07
TiO_2/Bent	19.96
10% GO/TiO_2	19.18
10% GO/TiO_2/Bent	33.59

Our study found that BET values of each catalyst are not related to the photocatalytic dye degradation activity. In Figure S2 and Table S1, we observed the order of BET surface area values from big to small is GO > 10% GO/TiO_2/Bent > TiO_2/Bent > 10% GO/TiO_2 > TiO_2. In Figure 6, the order of photocatalytic dye degradation activity from high to low is 10% GO/TiO_2/Bent > 10% GO/TiO_2 > TiO_2/Bent > TiO_2 > GO. So BET surface area is not the main influence factor here.

References

1. Jia, L.; Wang, D.H.; Huang, Y.X.; Xu, A.W.; Yu, H.Q. Highly durable n-doped graphene/CdS nanocomposites with enhanced photocatalytic hydrogen evolution from water under visible light irradiation. *J. Phys. Chem. C*, **2011,** *115*, 11466–11473.
2. Zhang, Y.; Tang, Z.R.; Fu, X.; Xu, Y.J. TiO_2–graphene nanocomposites for gas-phase photocatalytic degradation of volatile aromatic pollutant: is TiO_2–graphene truly different from other TiO_2–carbon composite materials? *ACS Nano* **2010,** *4*, 7303–7314.

© 2020 by the authors. Submitted for possible open access publication under the terms and conditions of the Creative Commons Attribution (CC BY) license (http://creativecommons.org/licenses/by/4.0/).
